# Delayed memory for complex visual stimuli does not benefit from distraction during encoding

**DOI:** 10.3758/s13421-023-01471-x

**Published:** 2023-09-29

**Authors:** Lea M. Bartsch, Philipp Musfeld

**Affiliations:** Department of Psychology, Cognitive Psychology Unit, University of Zurich, Zurich, Switzerland

**Keywords:** Working memory, Long-term memory, McCabe effect, Covert retrieval

## Abstract

The covert retrieval model (McCabe, *Journal of Memory and Language 58*(2), 480–494, 2008) postulates that delayed memory performance is enhanced when the encoding of memoranda in working memory (WM) is interrupted by distraction. When subjects are asked to remember stimuli for an immediate memory test, they usually remember them better when the items are presented without distraction, compared to a condition in which a distraction occurs following each item. In a delayed memory test, this effect has been shown to be reversed: Memory performance is better for items followed by distraction than without. Yet, this so-called McCabe effect has not been consistently replicated in the past. In an extensive replication attempt of a previous study showing the effect for complex visual stimuli, we investigated five potential boundary conditions of the predictions of the covert retrieval model: (1) Type of Stimuli (doors vs. faces), (2) type of distractor (pictures vs. math equations), (3) expectation about task difficulty (mixed vs. blocked lists), (4) memory load in WM (small vs. large), and (5) expectation about the long-term memory (LTM) test (intentional vs. incidental encoding). Across four experiments we failed to replicate the original findings and show that delayed memory for faces and other complex visual stimuli does not benefit from covert retrieval during encoding – as suggested as being induced by distractors. Our results indicate that the transfer of information from WM to LTM does not seem to be influenced by covert retrieval processes, but rather that a fixed proportion of information is laid down as a more permanent trace.

## Introduction

Working memory (WM) is understood as a capacity-limited store that holds information available for ongoing processing (Cowan, 2017; Oberauer, 2009). The system has been conceptualized as the gateway of perceptual information into long-term memory (LTM; Atkinson & Shiffrin, 1968), with the latter being the system for storing information more permanently with potentially unlimited capacity (Tulving, 1972). Within the classic model of Atkinson and Shiffrin (1968), the probability of information in the short-term store to be transferred into LTM was thought to be a function of the time for which that information was held in the short-term store. An alternative view was proposed by Craik and Lockhart (1972) who argued that the chance of establishing information in LTM depends on the depth with which it is processed rather than the duration for which it is held in a short-term store. To this day, the nature of the interaction of working and long-term memory is the subject of theoretical debates, with one open question being how information that is maintained in WM is transferred to LTM.

### The effect of distractors at encoding on delayed memory

Three common methods for investigating WM are simple span, complex span, and Brown-Peterson tasks. In the simple span, participants are presented with a list of items and asked to immediately recall them in forward serial order. In the complex span the items are interleaved with distractor tasks at encoding. Distractors that are commonly realized are reading sentences or evaluating arithmetic equations. Brown-Peterson tasks also require the recall of a short list of items yet here the retention interval is filled with a distractor processing task. What is typically observed is that performance in the immediate memory test is better in tasks without distraction (e.g., simple span), but worse for tasks including distraction (e.g., complex span, Brown-Peterson; Oberauer et al., 2015; Lewandowsky et al., 2010).

Work on comparing these different WM test paradigms with respect to their consequences for episodic LTM has resulted in contradicting results on whether (a) maintenance duration or (b) the way in which information is processed in WM determines a boost to delayed memory performance (McCabe, 2008; Souza & Oberauer, 2017; Zanto et al., 2016). In the first study comparing the long-term consequences of encoding and maintaining lists of words in a simple compared to a complex span task, McCabe (2008) observed that although immediate memory is better in simple than complex span tasks, delayed free recall for words used in the preceding WM tests was better for the items from complex span than simple span tasks. This *McCabe effect* has been replicated for words as memoranda (Loaiza & Mccabe, 2012) but not for non-words (Loaiza et al., 2015). The effect has initially been interpreted as evidence for a stronger involvement of LTM in complex than in simple span tasks in the following way: based on the Covert Retrieval Model, in complex span tasks, the distractors force people to temporarily outsource the words to LTM. Individuals then use part of the time during each distractor task to refresh the to-be-remembered words. This process of covertly retrieving the memoranda into WM leads to repeated and prolonged activation and thus better retrieval in the delayed test (McCabe, 2008).

Yet, subsequent work has shown that distractor processing is in fact not responsible for the boost of delayed free recall of the to-be-remembered verbal stimuli; rather it is the time for which memory materials are maintained (Souza & Oberauer, 2017). Complex span tasks usually entail a longer maintenance duration because the inter-item interval is extended to fit the distractor task. To control for the confounding variable of time, Souza and Oberauer (2017) implemented a *slow span task* in which the time of the inter-item interval was equated with the duration of the distractor task in the complex span task, with free time being inserted instead of distractors. When inter-item intervals in a simple span task were increased to the length of a complex span task in this way, delayed free recall for the slow span items was even better than for complex span items (Souza & Oberauer, 2017). Further, the McCabe effect was significantly attenuated in their study.

These results converge with the findings of Jarjat and colleagues (Jarjat et al., 2018), who tested delayed free recall of words that had served as memoranda in a complex span task in which they varied the number and the pace of distractor-task demands. The chance of delayed recall of a word increased with the total free time during which it had been maintained in the complex-span trial, which was determined by the word's list position, the number of distractors, and the free time in between distractors.

All of the above studies have investigated the consequences of maintenance in WM – and the distraction thereof – on episodic LTM with *verbal* stimuli, namely words or non-words. Together, they speak for a beneficial effect of maintenance duration rather than type of processing with respect to consequences for LTM. However, there is one study that calls into question whether this conclusion also holds for complex visual stimuli such as faces (Zanto et al., 2016). Across two experiments, Zanto and colleagues asked participants to remember grey-scaled faces (set size 1) for both an immediate old/new recognition test as well as a surprised delayed memory test. During the immediate memory test, they implemented a condition without distraction (i.e., a no-distraction condition), two conditions with longer retention intervals (i.e., like a slow span condition) and a condition with distraction (i.e., a like a Brown-Peterson task). They found that both increased retention intervals (like in a slow span task) as well as the insertion of a distractor face during the retention interval lead to worse memory in the immediate test. Yet, equivalent to the original McCabe effect and in line with the predictions of the Covert Retrieval Model (McCabe, 2008), incidental delayed memory was improved for faces initially encoded in both longer retention interval conditions as well as the distraction condition compared to the condition without distraction. These results stand in conflict with the pattern of results found in Souza and Oberauer (2017). In the present study we aimed (1) to replicate the findings of Zanto et al. (2016) to test the pattern's robustness and (2) to investigate five potential boundary conditions which could have caused diverging findings in the literature. Together this will allow insight into the interaction of WM and LTM.

### Potential boundary conditions of the effect of distraction on delayed memory

The studies of Zanto et al. (2016) and Souza and Oberauer (2017) resulted in conflicting patterns of results – yet they also differ in multiple key aspects, that could represent important mechanistic boundary conditions of the effect of distraction on maintenance in WM and thereby of the transfer of information from WM to LTM.

The key experimental parameters that differ between these two studies are the following: Type of stimuli (words vs. faces), type of distractor (pictures vs. math equations), expectation about task difficulty, memory load in WM, and expectation about the LTM test (intentional vs. incidental encoding). We discuss these in more detail in the following.

#### Type of stimuli

The most salient difference between the two above studies was the type of stimuli that had to be remembered. Although both the original McCabe Study as well as the failed replication by Souza and Oberauer (2017), were realized with words, the question remains whether complex visual stimuli such as the faces used in Zanto et al. (2016) would influence the covert retrieval of said memoranda during the presence of distractors or whether they would be prone to differential effects of free time.

In the past it has been shown that the capacity of visual WM is affected by the perceptual complexity of Stimuli (Eng et al., 2005), with lower capacity estimates for faces (~1) than for letters (~2.5). For words, WM capacity is commonly estimated to lie at around four (Cowan, 2001). This difference in capacity estimates could directly affect how much information can be maintained in WM – also in the presence of distractors – and thereby affect the degree of covert retrieval necessary. In case of an estimated WM capacity of 1 with regard to faces (Eng et al., 2005), the Covert Retrieval model would predict that already the presence of a single distractor following encoding of a single to-be-remembered face, would require the latter to be covertly retrieved from LTM. With words, instead, this necessity would be reduced.

Another possibility could be that faces are a special case even within visual working memory (Barry et al., 1998). Therefore, instead of trying to replicate the McCabe effect again with verbal stimuli as has been done (unsuccessfully) in several previous studies (Loaiza et al., 2015; Loaiza & Souza, 2021; Souza & Oberauer, 2017), here we aimed to investigate whether we can replicate Zanto and colleagues’ findings of a delayed memory benefit of distraction using faces and further extend their work by including a different type of complex visual stimuli, that lack the social and other special features of faces – namely pictures of doors (Baddeley et al., 2016).

#### Type of distractor

The next difference between the above studies refers to the type of distractor task implemented. In the original McCabe study (2008) as well as in Souza and Oberauer (2017), the distractor task entailed evaluating the correctness of a single mathematical equation in each inter-stimulus interval. While this means that the distractor was visually very different from the memoranda, it required WM to solve the equation as well as to execute a response via button press. Instead, in Zanto et al. (2017), the distractor entailed the presentation of another face, which the participants were instructed to ignore. This distractor was visually more similar to the memoranda, potentially leading to more visual interference; yet it required no response. Furthermore, the distractors were never presented as (false) memory probes in the immediate nor delayed test, thereby excluding the possibility of making a distractor-influenced mistake at retrieval. Therefore, our aim in the present study was to directly compare the effect of these two types of distractor tasks on their consequences for episodic LTM within a single experiment, realizing both a math-distraction and picture-distraction task.

#### Expectation about task difficulty

In the study by Souza and Oberauer (2017), participants could not predict whether the following trial would be a simple, complex, or slow span task, as this variable varied from trial to trial. Participants therefore could not prepare a certain strategy or expectation about the task difficulty before they were already doing it. In Zanto et al. (2016), the conditions were blocked, and participants could do all of the above. As has been suggested by Musfeld et al. (in press), expectations about the task difficulty can affect the transition of information from WM into LTM, with more difficult tasks having a facilitating effect on LTM. Therefore, we investigate this potential boundary condition of the effect of distraction on delayed memory, by comparing it in pure condition blocks to mixed blocks.

#### Memory load in working memory (WM)

The next difference between the above studies refers to the set size or memory load at encoding. In the original study, McCabe (2008) implemented set sizes of two, three, or four to-be-remembered words; in Souza and Oberauer (2017) participants encoded four or five words, whereas memory load in Zanto et al. (2016) was only a single face. There are two differences that arise from this. Firstly, at set sizes larger than one, the addition of a distractor after each item results in the classic structure of a complex span task. At set size one, the task becomes more of a Brown-Peterson style task, as technically the time interval following the first item is also the retention interval. Based on the predictions of the Covert Retrieval Model, the placement of the distractors – whether it is interleaving two memory items or one memory item and the memory test – should not make a difference, as long as the distractor task is sufficiently taxing and the capacity of the focus of attention is surpassed. Specifically, the model states that the distractors displace the to-be-remembered items from the focus of attention in working memory and force people to temporarily outsource the words to LTM (McCabe, 2008). As stated above, visual WM capacity for faces has been estimated to lie around 1 (Eng et al., 2005). Therefore, the Covert Retrieval model would predict that the presence of a single distractor following encoding of a single to-be-remembered face would already require the latter to be covertly retrieved from LTM.

Secondly, one can assume covert retrieval of a single item to be easier than having to retrieve up to five items following distraction,^1^ with the probability of retrieving a single item into WM being higher for set size 1 compared to set size 4 (or 5). The beneficial effect of distraction on LTM could therefore be larger for smaller set sizes. Instead, if the duration of maintenance drives the boost to delayed memory, set size should not have an effect on LTM (Bartsch et al., 2019). This is because the inter-stimulus intervals – and thereby the duration of maintenance – are the same for each individual item, independent of set size.

#### Expectation about the long-term memory (LTM) test (intentional vs. incidental encoding)

The previous studies on the McCabe effect differ in whether the participants initially were informed about the delayed memory test (intentional encoding) or not (incidental encoding). McCabe realized both an incidental delayed memory test (Exp. 1) and informed participants that there would be a delayed recall test (Exp. 2). Souza and Oberauer (2017) informed participants as well. Instead, Zanto et al. (2016) implemented a surprise delayed recognition test. Incidental learning consists of the information to be remembered being encoded into episodic memory, without an underlying intention (Craik & Tulving, 1975). Intentional learning, on the other hand, occurs when information is actively maintained in working memory through various strategies (Loaiza & Souza, 2021).

Decades of research have indicated that the intent to remember information has no effect on episodic LTM (e.g., Oberauer & Greve, 2021), yet this view was recently challenged by Popov and Dames (2022). They argued that the detection of an effect of intent in LTM commonly fails with previously used between-subject experimental designs. Using a new form of mixed-list within-subject designs, they did indeed show that episodic LTM is affected by the intent to remember information (Popov & Dames, 2022).

Why should the expectation about whether there will be a delayed memory test or not affect the occurrence of the McCabe effect? The covert retrieval model assumes that the McCabe effect occurs because participants engage in covert retrieval from LTM during WM tasks with distraction, which later leads to a boost on delayed memory performance. This presupposes that participants do not engage in more elaborative strategies in both tasks with and without distraction (McCabe, 2008). Yet, based on whether the subjects expect to have to recall the memoranda in a delayed test as well or not, might influence them to use elaborative strategies, and if they do, this could overshadow the effect of covert retrieval in WM tasks with distraction. We know only of a single study that has investigated the effect of intentional versus incidental learning on the McCabe effect directly: Loaiza and Souza (2021) found a general positive effect of intention on recall and showed that maintenance duration affects long-term recall primarily by actively maintaining information in WM. However, they did not find a consistent McCabe effect.

Here, we aimed to make use of the mixed-list design developed by Popov and Dames (2022) to directly investigate whether intent influences the effect of distraction in WM on delayed memory. To achieve this, in the present study we combined the design of Zanto et al. (2016) with the mixed-list design of Popov and Dames (2022).

### The present experiments

The goal of the present study was to replicate a previous study by Zanto et al. (2016) showing a beneficial effect of distraction on delayed memory for complex visual stimuli and to investigate the potential boundary conditions of the benefit – also known as the McCabe effect. To resolve the ambiguity from previous research on the effect – inspired by the concept of meta-studies (Baribault et al., 2018) – we asked whether differences in experimental parameters of previous studies (Zanto et al., 2016 vs. Loaiza & Souza, 2021; Souza & Oberauer, 2017) led to the diverging findings on the beneficial effect of distraction on delayed memory. These parameters included: (1) type of stimuli (doors vs. faces; Experiment 1), (2) type of distractor (pictures vs. math equations; Experiment 1), (3) expectation about task difficulty (pure vs. mixed blocks; Experiment 2), (4) set size (small vs. large; Experiment 3), and (5) expectation about the LTM test (intentional vs. incidental encoding; Experiment 4).

Across four experiments, we invited participants to complete a visual memory task online, in which pictures of grey-scaled faces were presented to them within the scope of simple span, slow span, and conditions with distraction. After an unrelated filler task, they completed a surprise delayed memory task for untested previously seen faces. If the McCabe effect was indeed subject to the said boundary conditions, we would expect to see better delayed memory performance for stimuli originally encoded within a condition with distraction in case the stimuli were faces, which were encoded at low set sizes, in case the distractors also were faces, in case the task conditions were blocked, and in case the encoding to memory was incidental.

## Experiment 1

### Method

#### Participants

We recruited a sample of *N* = 123 young volunteers from the student population from the University of Zurich. All participants had to be between 18 and 35 years of age (mean = 21.9 years). We ﻿chose an initial sample size of 120 participants because this was sufficient to detect medium-to-large effects in two-by-two factor within-subjects designs (type of stimuli and type of distractor). Psychology students from the University of Zurich received partial course credit for completing the 45-min experiment. Participants gave informed consent before the start of the experiment and were debriefed at the end. All experiments were carried out in agreement with the rules of the Ethics Committee of the Faculty of Arts and Sciences of the University of Zurich and did not require special approval.

#### Materials and procedure

**Fig. 1:**
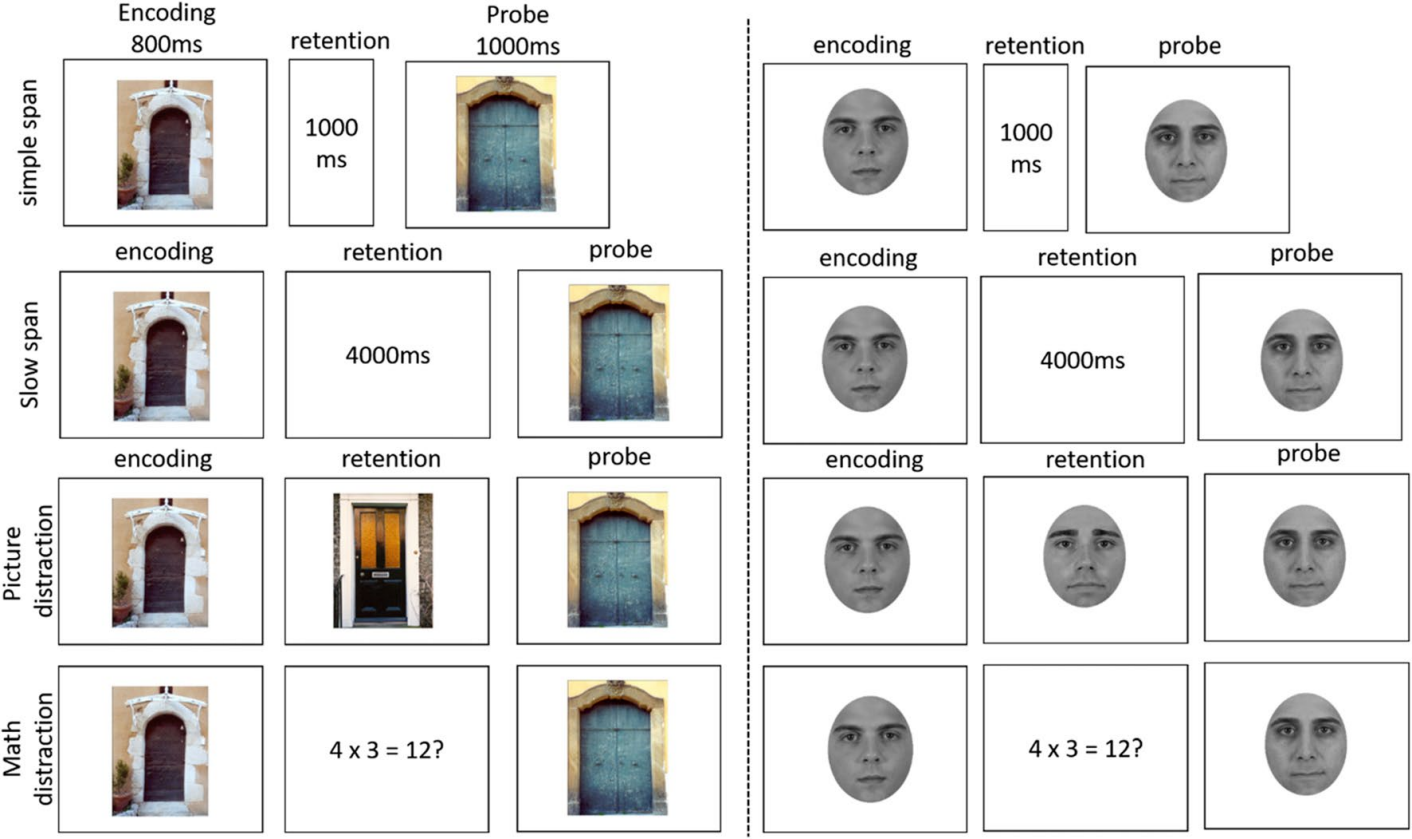
Illustration of the Immediate Memory Paradigm. Participants were shown a single stimulus (door or face) and were tested with an old/new recognition task

The experimental task was based on that used by Zanto et al. (2016), and was adapted to investigate our specific research question and be feasible for a purely behavioral experiment. Figure 1 provides an overview of the general procedure of Experiment 1. The experiment consisted of three phases: An immediate memory task, a delay filled with an unrelated task, and a final surprise delayed memory test. The immediate memory task consisted of encoding a single stimulus for an old/new recognition test. Further, we realized four different task conditions (simple span, slow span, picture-distraction, and a math-distraction condition) crossed with two different types of stimuli (faces and doors).

For the faces condition, the stimuli were drawn without replacement from a pool of 320 grey-scale faces taken from Goh et al. (2010). As in Zanto et al. (2016), identifying features like hair and clothes were removed. Faces were combined into pairs, so that lure probes for the immediate recognition test were matched in age and ethnicity. For the doors condition, stimuli were drawn without replacement from a pool of 200 pictures of doors from the “Doors for memory” database (Baddeley et al., 2016). Pictures in a vertical format were removed from the stimulus pool to make sure that all stimuli were presented in the same format.

Across eight practice trials, participants were familiarized with the conditions of the immediate memory task: the simple span, slow span, and both conditions with distraction. A fixation cross cued the beginning of a trial for 1,000 ms. In the simple span condition, a single face was presented for 1,000 ms followed by a 1,000-ms blank retention interval. In the slow span condition, a long retention interval of 4,000 ms was inserted, which matched the same amount of time in which the participants were presented with a distracting stimulus (e.g., another face, door or math equation) in the conditions with distraction. The picture-distraction condition matched the one realized in Zanto et al.’s study and consisted of presenting an irrelevant face during the retention interval. To make sure that participants encoded the distractor stimulus, they were asked to press the “space” bar as soon as the distractor stimulus came up. Visual feedback for a successful reaction was provided in form of a green point appearing at the bottom of the screen. The math-distraction condition required participants to indicate for a single math equation (e.g., 7 × 3 = 21?) via button press whether it was correct or not. This type of distractor was previously used in the original study by McCabe (2008) as well as in the study by Souza and Oberauer (2017). Again, visual feedback was provided by showing a green point for a correct response or a red point for a wrong response at the bottom of the screen. Immediate memory was tested the same way across all conditions: A probe appeared in the middle of the screen prompting subjects to indicate whether the face matched the one presented to them at the beginning of the trial via button press. Participants did not receive feedback regarding memory accuracy.

The immediate memory task was divided into 16 blocks, consisting of either seven or eight trials. Within each block, stimulus and task type were the same for every trial, and participants were informed about this before the beginning of each block. Therefore, participants were able to anticipate the difficulty of the task – equivalent to what was realized in Zanto et al. (2016). Each stimulus-task combination was realized across two blocks, once with seven and once with eight trials, leading to a total of 15 trials for each cell of the design. The order of blocks was pseudo-randomized so that each experimental condition had to be presented once before it was presented again. This made sure that no systematic bias was introduced between the time of presentation in the immediate memory phase and the time of testing in the delayed memory.

After a delay filled with an unrelated task, the final surprise delayed memory test consisted of the presentation of 144 stimuli (faces and doors), which participants were asked to classify as either "definitely old," "probably old," "probably new," or "definitely new." There was no time limit for the classification. The stimuli shown comprised 50% stimuli from the immediate memory phase and 50% stimuli that had never been shown before. The stimuli from the immediate memory phase were only stimuli that had not been tested in the immediate memory test.

#### Data analysis

For analyzing the data, we used Bayesian hierarchical signal detection models to estimate participants’ memory performance in terms of d-prime. D-prime is a measure of participants’ ability to discriminate between old and new items, which is independent from the decision criteria, i.e., the general tendency for selecting the old or new response (Green & Swets, 1966; Wixted, 2020). Because the immediate and delayed memory test varied in their response format (“old”/“new” recognition in the immediate test vs. “sure old” / “probably old” / “probably new” / “sure new” in the delayed test), we fitted separate models to the data from both tasks.

For the immediate memory test, we modeled the probability of an “old” response by a probit regression model as follows:

$${\displaystyle \begin{array}{c}{n}_{old}\mid {n}_{responses}\sim Binomial\left({p}_{old}\right)\\ {}{p}_{old}=\phi \left({\beta}_0+{\beta}_1\ast isold\right)\end{array}}$$

In this specification of the model, *β*_0_ reflects the negative decision criterion (*-c*), whereas *β*_1_ reflects d-prime. By applying a probit-link (*ϕ*) on the linear model term, the outcome is transformed to a probability reflecting the chance of responding “old,” given the presented probe stimulus (Vuorre, 2017). We then added *task* and *stimulus material* as predictor to the linear model term, to estimate the effect of our experimental conditions on participants’ d-prime.

For the delayed memory test, the model specification was similar, but with the difference that we used an ordered probit regression model to account for the four different ordered outcome categories. The four different outcome categories can be interpreted as four different levels of confidence. To model this data in a signal detection framework, three different response criteria need to be estimated, which determine at which point the next confidence level is chosen. Therefore, instead of modeling the probability of an “old” response, we modeled the cumulative probability of responding with category *k* or less as follows:

$${\displaystyle \begin{array}{c}\ {y}_i\sim Cumulative\left(p\left({y}_i\le {k}_i\right)\right)\\ {}p\left({y}_i\le {k}_i\right)=\phi \left({\beta}_{0_k}+{\beta}_1\ast {isold}_i\right)\end{array}}$$

Here, separate intercepts (response criteria *c*) are estimated for each response category *k*, whereas *β*_1_ still reflects d-prime, as the participant’s ability to discriminate between “old” and “new” items. We again added *task* and *stimulus material* as predictors to the linear model term to estimate their effect on participants’ d-prime.

All models were fitted in R (*v. 4.2.1,* R Core Team, 2022) together with the R-packages *brms* (Bürkner, 2017), and included random participant effects on all estimated parameters (Barr et al., 2013; Oberauer, 2022).

To quantify the evidence for differences in d-prime between the experimental conditions (e.g., difference in d-prime between the simple and complex span condition), we estimated Bayes Factors (BFs) for all pairwise comparisons of interest. BFs were approximated using the Savage-Dickey Density Ratio (Wagenmakers et al., 2010) between the estimated posterior distribution and the exact prior distribution. A BF larger than 1 gives evidence for an effect (i.e., in favor of a difference ≠ 0), a BF_10_ lower than 1 provides evidence against an effect and hence evidence for the null hypothesis. We considered BFs > 3 as substantial evidence for one hypothesis over the other, and regarded BFs < 3 as inconclusive (Kass & Raftery, 1995).

We used normal priors with mean = 0 and SD = 1 for all effect variables in the model, as these reflect reasonable assumptions about effect sizes in a signal detection framework. To ensure robustness of our results from the prior, we performed prior sensitivity analyses and varied the scale of the prior at standard deviations of 0.5, 1, 1.5, and 2. In the Results sections, we always report the value for SD = 1 together with the range of BFs obtained in the prior sensitivity analysis.

## Results

**Fig. 2:**
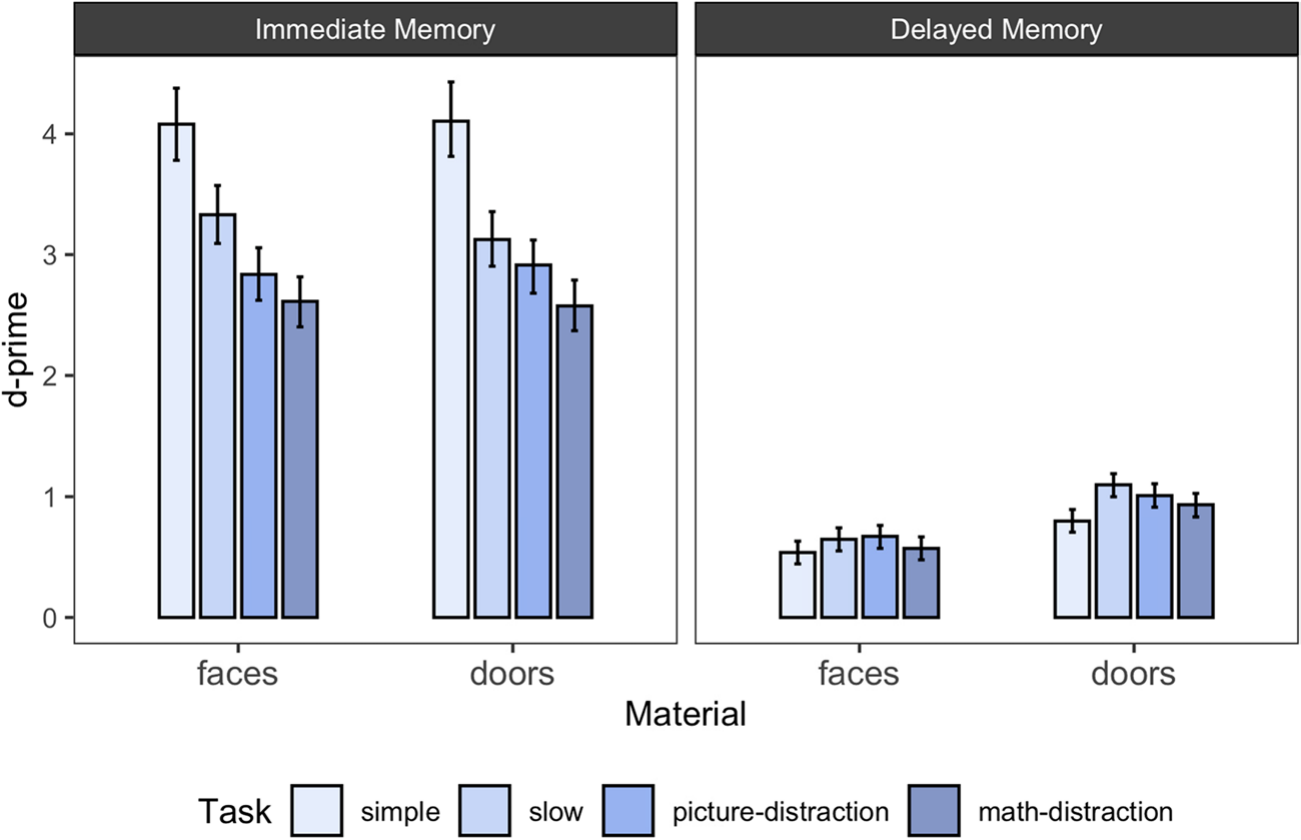
Immediate and delayed memory performance across the different stimulus types and task conditions in Experiment 1. *Note.* Error bars reflect 95% highest density intervals of the estimated posterior distributions for d-prime

The results of Experiment 1 are presented in Fig. 2 and Table 1*.* Figure 2 shows the posterior estimates of d-prime for each experimental condition together with the 95% highest density interval. Table 1 shows the BFs for all pairwise comparisons between the different task conditions. In the following, we report the results of Experiment 1 in light of two research questions: (1) Is there evidence for a McCabe effect for remembering faces and doors? (2) Is the McCabe effect influenced by the type of distractor?

**Table 1:** Results of our Bayes Factor (BF) analysis for all pairwise comparisons between the different task conditions. We report BFs together with their range obtained in the prior sensitivity analysis as well as the difference in d-primes between the two compared conditions

	Immediate memory		Delayed memory	
Contrasts	BF_10_ [Prior Range]	*Δd’* [95% HDI]	BF_10_ [Prior Range]	*Δd’* [95% HDI]
Faces				
Simple vs. Slow	8.06 × 10^2^ [2.53 – 16.92 × 10^2^]	0.75 [0.43 – 1.12]	0.23 [0.11 – 0.47]	-0.11 [-0.21 – -0.00]
Simple vs. Picture-Distraction	2.40 × 10^8^ [0.10 – 10.92 × 10^8^]	1.24 [0.92 – 1.58]	0.65 [0.32 – 1.31]	-0.13 [-0.24 – -0.03]
Simple vs. Math-Distraction	15.68 × 10^9^ [0.94 – 214.35 × 10^9^]	1.46 [1.14 – 1.78]	0.03 [0.01 – 0.06]	-0.03 [-0.14 – 0.07]
Slow vs. Picture-Distraction	32.74 [17.49 – 57.72]	0.50 [0.22 – 0.78]	0.03 [0.01 – 0.05]	-0.02 [-0.13 – 0.08]
Slow vs. Math-Distraction	14.92 × 10^3^ [1.98 – 23.57 × 10^3^]	0.71 [0.45 – 0.98]	0.07 [0.04 – 0.15]	0.08 [-0.03 – 0.18]
Picture-Distraction vs. Math-Distraction	0.26 [0.13 – 0.49]	0.22 [-0.04 – 0.46]	0.15 [0.08 – 0.31]	0.10 [-0.00 – 0.20]
Doors				
Simple vs. Slow	4.62 × 10^4^ [1.63 – 13.61 × 10^4^]	0.98 [0.65 – 1.33]	39.99 × 10^3^ [2.88 – 39.99 × 10^3^]	-0.30 [-0.40 – -0.20]
Simple vs. Picture-Distraction	10.66 × 10^6^ [0.38 – 16.45 × 10^6^]	1.19 [0.87 – 1.56]	41.38 [22.80 – 89.77]	-0.21 [-0.31 – -0.11]
Simple vs. Math-Distraction	15.64 × 10^9^ [0.13 – 126.52 × 10^9^]	1.53 [1.20 – 1.86]	0.64 [0.31 – 1.34]	-0.14 [-0.24 – -0.03]
Slow vs. Picture-Distraction	0.23 [0.11; 0.45]	0.21 [-0.06 – 0.47]	0.10 [0.05 – 0.20]	0.09 [-0.02 – 0.19]
Slow vs. Math-Distraction	370.79 [114.53 – 792.10]	0.55 [0.30 – 0.80]	2.77 [1.45 – 5.95]	0.17 [0.06 – 0.27]
Picture-Distraction vs. Math-Distraction	1.65 [0.85 – 3.22]	0.34 [0.09 – 0.60]	0.07 [0.03 – 0.14]	0.08 [-0.03 – 0.18]

All data and analysis scripts can be accessed on the Open Science Framework (https://osf.io/rwa3s/).

### Performance in the distractor task

To make sure that participants attended to the distractor task in the two distraction conditions, we tracked participants reactions to the distractors during the experiment. Participants were excluded from the experiment if they did not react to either the picture or the equation in the retention interval more than six times (which corresponds to 10% of distraction trials). Overall, participants reacted to 95% of distractors in the picture-distraction task and 98% of distractors in the math-distraction task. The average accuracy in the equation distractor task was 91% (*SD* = 2.8), showing that participants did well in the task. The distractor tasks differed in the amount of time participants took to react to the distractor, with an average of *M*_*RT*_ *=* 493.16 ms (*SD*_*RT*_ = 401.99 ms) in the picture-distraction task and an average of *M*_*RT*_ *=* 1726.14 ms (*SD*_*RT*_ = 655.91 ms) in the math-distraction task.

#### Is there evidence for a McCabe effect for remembering faces and doors?

To evaluate the presence or absence of a McCabe effect, we examined for both stimulus materials whether there was evidence for better immediate memory performance in the simple compared to the distraction tasks, as well as the opposite pattern in the delayed memory task (= a pattern representing the McCabe effect). We first focus on the comparison to the picture-distraction condition, before comparing it to performance in the math-distraction task. All BFs, Bayesian Sensitivity Ranges, and descriptive difference in d-primes can be found in Table 1.

In the immediate memory test, our results show the typical pattern of better memory performance in the simple span task compared to the picture-distraction task. This was true for both faces and doors. Notably, different from Souza and Oberauer (2017), our results indicated a detrimental effect of free time in the immediate memory test.

In the delayed memory test, we observed a pattern that is at a first glance consistent with the McCabe effect: For both faces and doors, the estimated d-primes were descriptively smaller for the simple span condition compared to the picture-distraction condition. However, BF analysis revealed credible evidence only in favor of a difference for doors. For faces, evidence was inconclusive but tended *against* the presence of a difference (see Table 1).

In order to discriminate whether the benefit on the delayed memory performance that we saw for door-stimuli in the picture-distraction versus simple span condition was attributable to the presence of distractors, or whether the longer maintenance duration led to better performance, we compared performance to the slow span task next. Indeed, equivalent to Souza and Oberauer (2017), we also found a beneficial effect of the slow span condition compared to the simple span condition and critically, delayed memory performance in the slow span condition did not differ from performance in the picture-distraction condition (see Table 1 for all BFs). Taken together, the benefit of distraction versus simple span performance we found for doors – but not for faces – is better explained by the extended maintenance duration and not by the distraction itself.

#### Is the McCabe effect influenced by the type of distractor?

Next, we turned to the comparison of the two conditions with distraction, to investigate whether the occurrence of the McCabe effect was affected by the distractor being similar to the memoranda (picture-distraction task; as in Zanto et al., 2016), or not. As seen in Fig. 2, both immediate and delayed memory performance was slightly worse in the math-distraction task compared to the picture-distraction task. However, BF analyses mostly provided evidence against a credible difference for both memory tests and materials.

To evaluate the McCabe effect in the math-distraction condition, we again compared it to the simple span condition in the immediate and delayed memory test. Immediate memory performance was clearly better in the simple compared to the math- distraction task for both materials. However, in the delayed memory test, we found evidence against a difference for faces, and evidence remained inconclusive for doors. Therefore, when evaluated on the math-distraction span condition, data of neither of the stimulus materials resulted in conclusive evidence for the pattern predicted by the Covert Retrieval Model and known as the McCabe effect.

### Interim discussion: The effect of distraction on delayed memory as a function of type of stimuli and distractors

In Experiment 1, we aimed to replicate the findings of Zanto et al. (2016), who found better delayed memory for faces encoded in a condition with distraction, in which the distractors were also faces, compared to faces encoded in a simple span task. To generalize the findings to other complex visual material, we extended the design by another stimulus-material (doors). Further, to directly investigate the potential effect of design choices to previous studies on the McCabe effect we also extended the design by another distraction condition, in which distractor stimuli were math equations. Overall, our findings did not replicate the results and conclusions obtained by Zanto et al. (2016). Only for the door stimuli did we find conclusive evidence for better delayed memory performance in a picture-distraction task compared to the simple span task. Critically, the comparison to the time-matched slow-span condition indicates that this benefit on delayed memory performance can be attributed to the extended encoding time and not to covert retrieval sparked by the distraction itself.

Furthermore, this effect was not present when comparing the simple span task to another distraction condition, in which we presented math equations instead of additional pictures. What could have caused this? One notable difference between the two distraction conditions is the time participants need for reacting to the distractor: Participants reacted much quicker to the picture in the retention interval than to the math equation. This suggests that the picture-distractor condition allowed more undistracted free time than the math-distraction, thereby making it more similar to the slow span condition, in which the time in the retention time is equated with the time in the distraction condition. This further supports our conclusion that the beneficial effect of the complex-distraction condition on delayed memory performance is caused by the extended amount of free time rather than through the presence of the distractor task itself (see also Souza & Oberauer, 2017).

Overall, our results do not replicate the findings by Zanto et al. (2016) and are rather consistent with the conclusion from Souza and Oberauer (2017): beneficial effects on delayed memory performance are best explained by the time for processing than a covert retrieval mechanism. Next, we turn to investigating whether the expectation about task difficulty is a boundary condition of the beneficial effect of distraction on delayed memory performance.

## Experiment 2

### Method

#### Participants

We recruited an independent sample of N = 65 young volunteers from the student population from the University of Zurich. All participants had to be between 18 and 35 years of age (mean = 21.8 years). The study took approximately 45 min and participants were compensated with partial course credit.

#### Materials and procedure

The general task and procedure were the same as in Experiment 1 except for the following changes: In order to manipulate the expectation about task difficulty, the conditions of simple, slow, and distraction conditions were either presented in mixed blocks, in which the condition type could vary from trial to trial, or in pure blocks, in which only one task type was realized throughout. In pure blocks, participants could build up an expectation about whether the following trial would be difficult (distraction condition) or not and could potentially adapt their strategies and efforts prior to encoding. The set-up of pure blocks is the same as in the Zanto et al. (2016) study.

We realized six pure and six mixed blocks of seven to eight trials, which led to a total of 15 trials per experimental condition. Again, the order of conditions in pure blocks was pseudorandomized so that each condition (simple span, slow span, or distraction condition) was realized once before a second block of only that task condition was repeated. In the *mixed* blocks, the three task conditions were distributed equally over the seven to eight trials of a block, so that at the end, also 15 trials per experimental condition were realized and distributed equally over the experiment.

Because participants were not able to anticipate the task condition of a trial in the mixed blocks, distractor faces in the distraction condition were additionally surrounded with a red frame. This reduced the possibility to confuse the distractor stimulus for the probe stimulus.

Finally, we opted to only present faces as stimuli and only realize the picture-distraction task, in order to stay as close as possible to the paradigm from Zanto et al. (2016).

#### Data analysis

We analyzed the data equivalently to Experiment 1, with the only difference being that we estimated the effect of the blocking type (pure vs. mixed blocks) on d-prime.

### Results

**Fig. 3:**
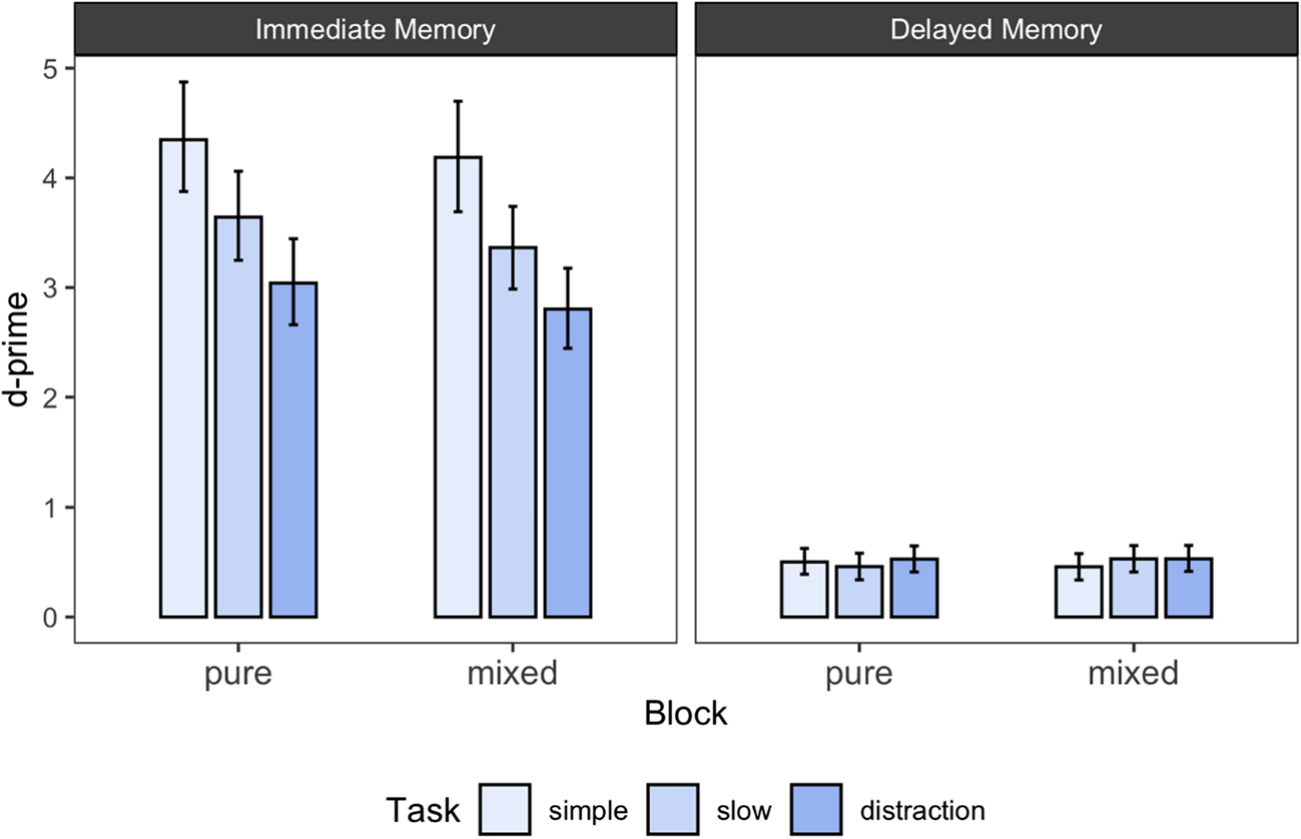
Immediate and delayed memory performance across mixed and pure blocks as well as task. *Note.* Error bars reflect 95% highest density intervals of the estimated posterior distributions for d-prime

The results of Experiment 2 are presented in Fig. 3 and Table 2. Figure 3 shows the posterior estimates of d-prime for each experimental condition together with the 95% highest density interval. Table 2 shows the BFs for all pairwise comparisons between the conditions in Experiment 2, as well as the descriptive difference in d-primes

**Table 2:** Results of our Bayes Factor (BF) analysis for all pairwise comparisons between the different task conditions. We report BFs together with their range obtained in the prior sensitivity analysis as well as the difference in d-primes between the two compared conditions

	Immediate memory		Delayed memory	
Contrasts	BF_10_ [Prior Range]	*Δd’* [95% HDI]	BF_10_ [Prior Range]	*Δd’* [95% HDI]
Pure Blocks				
Simple vs. Slow	5.03 [2.73 – 8.35]	0.71 [0.19 – 1.25]	0.04 [0.02 – 0.08]	0.04 [-0.09 – 0.18]
Simple vs. Distraction	2.84 × 10^4^ [1.08 – 5.49 × 10^4^]	1.31 [0.79 – 1.81]	0.03 [0.02 – 0.07]	-0.02 [-0.16 – 0.11]
Slow vs. Distraction	4.34 [2.21 – 9.36]	0.60 [0.16 – 1.03]	0.05 [0.03 – 0.11]	-0.07 [-0.21 – 0.07]
Mixed Blocks				
Simple vs. Slow	19.44 [12.89 – 32.16]	0.82 [0.29 – 1.33]	0.06 [0.03 – 0.12]	-0.07 [-0.21 – 0.07]
Simple vs. Distraction	3.75 × 10^4^ [3.75 – 24.61 × 10^4^]	1.38 [0.90 – 1.91]	0.06 [0.03 – 0.11]	-0.07 [-0.21 – 0.06]
Slow vs. Distraction	5.54 [2.81 – 10.58]	0.56 [0.17 – 0.95]	0.03 [0.02 – 0.06]	0.00 [-0.13 – 0.13]

To evaluate the presence of a McCabe effect, we examined for both blocking conditions whether there was evidence for better immediate memory performance in the simple span compared to distraction condition, as well as the opposite pattern in the delayed memory task (= a pattern representing the McCabe effect).

As in Experiment 1, results from the immediate memory test showed the typical pattern of better memory performance following a simple span task compared to distraction condition. This was true for both the pure and the mixed block condition. However, across both blocking types we found clear evidence against a difference between simple span and distraction condition in the delayed memory test (see Table 2 for all BFs). Therefore, our results neither replicate the findings by Zanto et al. (2016), nor the original McCabe effect (McCabe, 2008). Furthermore, we can conclude that the expectation of the task difficulty had no influence on this finding.

Next, we turned to the effects of extended free time on immediate and delayed memory performance – operationalized by the slow span condition. For immediate memory performance, we again found evidence for a detrimental effect of extended free time in the slow compared to the simple span condition. For delayed memory performance, however, there was neither a difference to performance in the simple span, nor to the distraction condition. Thus, in contrast to Experiment 1, there was no benefit of extended free time on delayed memory performance.

### Interim discussion: The effect of distraction on delayed memory as a function of expectation about task difficulty?

Here, we again aimed to replicate the findings of Zanto et al. (2016), who found better delayed memory for faces encoded with distraction in which the different task types were realized in pure blocks. Our results do not replicate such a McCabe effect, neither for the original pure task blocks, nor mixed blocks. With this we replicate previous failed attempts to replicate the original McCabe effect (Loaiza et al., 2015; Loaiza & Souza, 2021; Souza & Oberauer, 2017). Therefore, our results speak against the expectation about task difficulty being a boundary condition of the Covert Retrieval Model.

In contrast to findings by Souza and Oberauer (2017), our findings also showed no effect of extended free time (slow span condition) on delayed memory performance. Next, we turn to investigating whether set size at encoding is a boundary condition of the predictions of the Covert Retrieval Model.

## Experiment 3

### Method

#### Participants

We recruited an initial independent sample of *N* = 57 young volunteers from the student population from the University of Zurich. These participants were compensated with partial course credits. Because evidence for our research question was still inconclusive, we collected another 20 participants on *Prolific*, which is possible in a Bayesian analysis framework in case of ambiguous evidence (Rouder, 2014). Participants on *Prolific* received £6.75 (~USD 8). The final sample consisted of *N* = 77 participants between 18 and 35 years of age (mean = 24.3 years).

#### Materials and procedures

The general task and procedure were the same as in Experiment 2 except the following changes: In order to manipulate WM load at encoding we varied the set size of to-be-remembered faces between 1 and 3. To increase discriminability between to-be-remembered and distractor faces in the picture-distraction task, distractor faces were presented with a red frame. Note that at set size 3, the distractors are presented interleaving the memory items, making this task a classic complex span task. Yet, as outlined above, the location of the distraction task should be irrelevant to whether it displaces the information from WM, as long as the capacity of the focus of attention is exceeded.

Again, the experiment was divided into 12 blocks of seven to eight trials, with a total of 15 trials per experimental condition. All blocks were pure (like in Zanto et al. 2016) and realized one combination of task condition (simple, slow span, and distraction) and set size (1 vs. 3) throughout. There were two blocks for each condition combination and the order of blocks was pseudorandomized so that each condition combination was presented once before it was repeated.

Because of the higher amount of presented stimuli in the set size 3 condition, we added an additional set of 60 faces drawn from the Chicago Face Database (Ma et al. 2015). The new faces were edited in the same way as the original ones and combined into pairs which were matched in gender and ethnicity. This resulted in a final stimulus set of 380 faces.

#### Data analysis

We analyzed the data equivalently to Experiment 1, with the only difference that we estimated the effect of set size on d-prime.

### Results

**Fig. 4:**
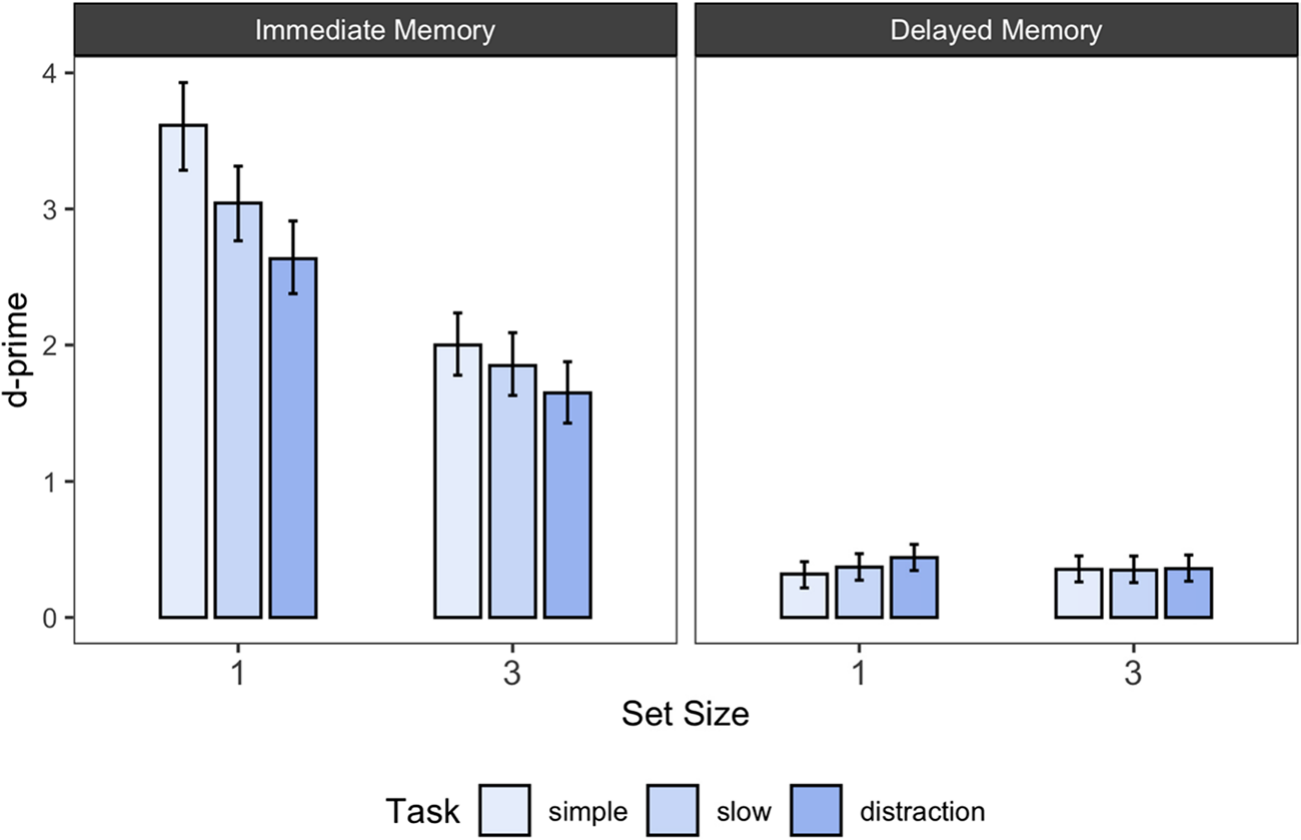
Immediate and delayed memory performance across set size 1 and 3 as well as task conditions in Experiment 3. *Note.* Error bars reflect 95% highest density intervals of the estimated posterior distributions for d-prime

The results of Experiment 3 are shown in Fig. 4 and Table 3. Figure 4 shows the posterior estimates of d-prime for each experimental condition together with the 95% highest density interval. Table 3 shows the BFs for all pairwise comparisons between the different task conditions, as well as the descriptive difference in d-primes. In the following, we report the results of Experiment 3 in light of two research questions: (1) Is there evidence for a McCabe effect? (2) Is the McCabe effect influenced by the set size at encoding?

**Table 3:** Results of our Bayes Factor (BF) analysis for all pairwise comparisons between the different task conditions. We report BFs together with their range obtained in the prior sensitivity analysis as well as the difference in d-primes between the two compared conditions

	Immediate memory		Delayed memory	
Contrasts	BF_10_ [Prior Range]	*Δd’* [95% HDI]	BF_10_ [Prior Range]	*Δd’* [95% HDI]
Set Size 1				
Simple vs. Slow	7.25 [3.61 – 12.29]	0.57 [0.21 – 0.97]	0.04 [0.02 – 0.08]	-0.05 [-0.17 – 0.08]
Simple vs. Distraction	3.02 × 10^3^ [3.02 – 58.57 × 10^3^]	0.98 [0.59 – 1.35]	0.19 [0.09 – 0.37]	-0.12 [-0.24 – 0.00]
Slow vs. Distraction	1.26 [0.63 – 2.48]	0.41 [0.08 – 0.76]	0.06 [0.03 – 0.11]	-0.07 [-0.20 – 0.05]
Set Size 3				
Simple vs. Slow	0.12 [0.06 – 0.23]	0.15 [-0.13 – 0.42]	0.03 [0.01 – 0.06]	0.01 [-0.12 – 0.13]
Simple vs. Distraction	1.75 [0.90 – 3.53]	0.36 [0.10 – 0.64]	0.03 [0.01 – 0.06]	-0.01 [-0.12 – 0.13]
Slow vs. Distraction	0.19 [0.09 – 0.37]	0.20 [-0.07 – 0.50]	0.03 [0.01 – 0.06]	-0.01 [-0.13 – 0.11]

To evaluate the presence of a McCabe effect, we examined for both set sizes whether there was evidence for better immediate memory performance in the simple span compared to the distraction condition, as well as the opposite pattern in the delayed memory task (= a pattern representing the McCabe effect).

For immediate memory performance, we again descriptively found the typical pattern of better memory performance in the simple span compared to the distraction condition. However, BF analyses showed only credible differences between the simple and the distraction condition for set size 1; for set size 3, memory performance was not only generally reduced, but also the evidence for a difference between the simple span and the distraction condition remained inconclusive (see Table 3 for all BFs).

For the delayed memory test, we found clear evidence *against* a difference between the simple span and the distraction condition – for both set size conditions. In contrast to the immediate memory test, there was also no general difference in performance between the two set sizes.

Taken together, these results stand in contrast to findings of Zanto et al. (2016) as well as the predictions of the Covert Retrieval Model (McCabe, 2008).

With regard to the slow span condition, we again found a detrimental effect on immediate memory performance for set size 1, but not for set size 3. For delayed memory performance, the analysis neither credibly supported a difference between the slow and the simple span condition, nor between the slow span and the distraction condition. Thus, we again did not find a beneficial effect of extended free time on delayed memory performance.

### Interim discussion: The effect of distraction on delayed memory as a function of set size at encoding?

In previous studies on the McCabe effect different levels of WM load at encoding had been realized. While a recent study showing the effect realized a rather small set size of remembering a single face (Zanto et al., 2016), previous failed replications opted for larger set sizes (e.g., six words; Souza & Oberauer, 2017). Here, we investigated within a single experiment whether the initial WM load would affect the occurrence of the McCabe effect. Our results show that the factor had no influence and that again, we did not replicate the effect in neither set size. The latter further supports that the lack evidence for a McCabe effect in the previous experiments is not due to the distraction condition being more similar to a Brown-Peterson than a complex span task. Both distractors interleaving items (at set size 3) and distractors in the retention interval (at set size 1) should have displaced memoranda from WM to LTM, thereby strengthening their LTM representations – yet we found evidence for neither.

Further, set size had no effect on LTM, replicating previous work (Bartsch et al., 2019). Next, we turn to investigating whether the intent to remember is a boundary condition of the of the predictions of the Covert Retrieval Model.

## Experiment 4

### Method

#### Participants

We recruited an initial independent sample of *N* = 57 young volunteers from the student population from the University of Zurich. These participants were compensated with partial course credits. Because evidence for our research question was still inconclusive, we collected another 43 participants on *Prolific*, resulting in a total sample size of *N* = 120. All participants were between 18 and 35 years of age (mean = 24.4 years). Participants on *Prolific* received £6.75 (~USD 8) for their participation.

#### Materials and procedures

**Fig. 5:**
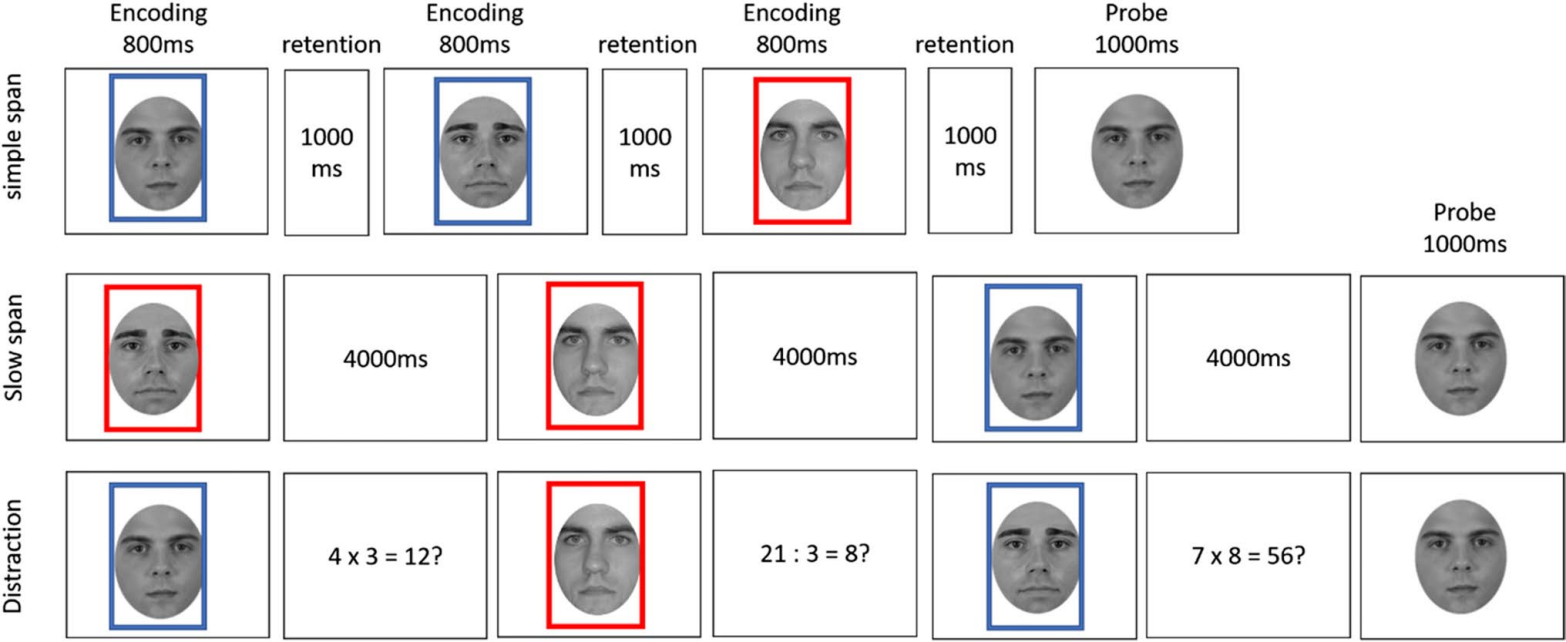
Illustration of the immediate memory paradigm of Experiment 4. Participants were shown three stimuli (faces), the color of the frame indicated whether it was a to-be-remembered face or not and were tested with an old/new recognition task

The general task and procedure were the same as in the previous experiments except the following changes: In order to manipulate intent to remember within-subject we implemented a task equivalent to a recent study on the effects of intent on memory performance (Dames & Popov, 2022): Specifically, subjects were presented with three different faces in each task, one after the other, which they had to rate as likeable or unlikeable without a time limit. This orienting task ensured that all stimuli were encoded, independently of the intent manipulation. This intent manipulation entailed that each face was surrounded by either a blue or red frame. Subjects were instructed to remember only faces that had a frame in one of the two colors, which was counterbalanced across participants (see Fig. 5). Thus, the stimuli were separated into to-be-remembered and to-be forgotten items within each participant. The latter represented the incidental learning condition. Of the three faces in each trial, at least one was a to-be-remembered stimulus and at least one was a forget stimulus. The third stimulus was always equally likely to be either a to-be-remembered or a forget stimulus.

In the simple and slow span condition, after rating each face, 1,000 ms or 4,000 ms passed until the next face appeared, respectively. In the distraction condition, a single math equation (e.g., 7 × 3 = 21?) was presented after each face and participants were required to indicate whether it was correct or not, via button press. We used a math equation for the distraction condition in this experiment because the design already required participants to encode additional faces which were not to-be-remembered. After the presentation of the third stimulus, there was a retention interval of 1,000 ms, followed by the immediate test. Here, the subjects were presented with a probe face, which never was a to-be-forgotten face. We realized six blocks of ten trials – two blocks of each of the three conditions (simple, slow span, distraction) – leading to a total of 20 trials per experimental condition.

Finally, in the LTM test half of the probe stimuli from the immediate memory phase were to-be-remembered items and the other half were to-be-forgotten items.

Again, due to the increased amount of presented faces in this experiment, we added the additional set of 60 faces drawn from the Chicago Face Database (Ma et al., 2015), which were also used for Experiment 3.

#### Data analysis

We analyzed the data similarly to Experiment 1, with the only difference that we estimated the effect of the intention to remember on d-prime. However, this was only possible for the delayed memory test because to-be-forgotten items were never tested in the immediate test. Therefore, the model for the immediate memory performance only considered the task condition as a predictor on d-prime.

### Results

**Fig. 6:**
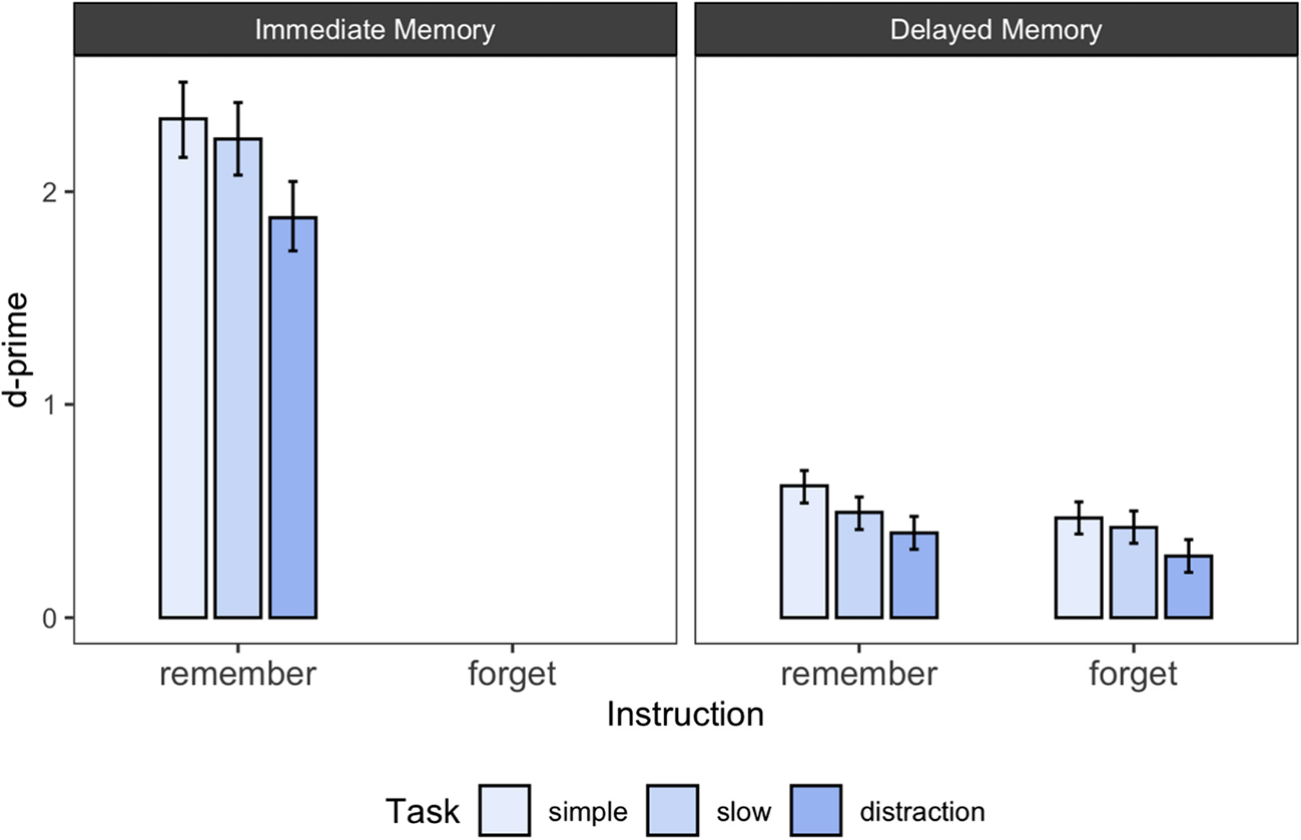
Immediate and delayed memory performance across instructions to remember or forg*et as well as* t*ask* c*onditions in Experiment 4. Note. Error bars reflect 95% highest density intervals of the estimated posterior distributions for d-prime*

The results of Experiment 4 are presented in Fig. 6 and Table 4. Figure 6 shows the posterior estimates of d-prime for each experimental condition together with the 95% highest density interval. Table 4 shows the BFs for all pairwise comparisons between the different task conditions, as well as the descriptive difference in d-primes. In the following, we report the results of Experiment 4 in light of two research questions: (1) Is there evidence for a McCabe effect? (2) Is the McCabe effect influenced by the intent to remember at encoding?

**Table 4:** Results of our Bayes Factor (BF) analysis for all pairwise comparisons between the different task conditions. We report BFs together with their range obtained in the prior sensitivity analysis as well as the difference in d-primes between the two compared conditions

	Immediate memory		Delayed memory	
Contrasts	BF_10_ [Prior Range]	*Δd’* [95% HDI]	BF_10_ [Prior Range]	*Δd’* [95% HDI]
Remember				
Simple vs. Slow	0.09 [0.04 – 0.18]	0.09 [-0.10 – 0.29]	1.27 [0.68 – 2.50]	0.13 [0.04 – 0.21]
Simple vs. Distraction	1.08 × 10^3^ [1.08 – 7.71 × 10^3^]	0.46 [0.28 – 0.65]	10.43 × 10^2^ [4.10 – 35.72 × 10^2^]	0.22 [0.14 – 0.31]
Slow vs. Distraction	147.90 [90.50 – 332.02]	0.37 [0.19 – 0.54]	0.27 [0.12 – 0.49]	0.10 [0.01 – 0.18]
Forget				
Simple vs. Slow			0.03 [0.02 – 0.07]	0.04 [-0.04 – 0.13]
Simple vs. Distraction			40.39 [40.39 – 147.82]	0.18 [0.09 – 0.26]
Slow vs. Distraction			2.86 [1.24 – 4.86]	0.13 [0.05 – 0.22]

We examined whether there was evidence for better immediate memory performance in the simple span compared to the distraction condition, as well as the opposite pattern in the delayed memory task (= a pattern representing the McCabe effect). For the immediate memory test, we could only consider faces which were encoded with the intent to remember, as to-be-forgotten faces were never tested.

For the immediate memory task, we again found evidence for the expected pattern of better memory performance in the simple span compared to the distraction condition (see Table 4 for all BFs). Similar to the larger set size condition in Experiment 3, immediate memory performance was also generally reduced compared to our previous experiments due to the higher set size at encoding.

For the delayed memory performance, we found evidence for a credible difference between the simple span and the distraction condition. However, opposite to the predictions of the McCabe effect, delayed memory performance was *better* for the simple span condition compared to the distraction condition. Thus, we again did not replicate the findings by Zanto et al. (2016), but found evidence for an effect in the opposite direction.

Next, we analyzed the effect of the intent to remember on delayed memory performance. Here, we only found evidence for a beneficial effect of intent in the simple span condition (BF_10_ = 6.64 [3.54; 17.60]), but not for the slow (BF_10_ = 0.07 [0.04; 0.14]), or the distraction condition (BF_10_ = 0.42 [0.22; 0.83]).

### Interim discussion: McCabe effect as a function of intent to remember?

Here, in Experiment 4, we investigated within a single experiment whether the intent to remember information in WM would affect the occurrence of the beneficial effect of distraction on delayed memory performance. Our results show that the factor had no influence and that again, we did not replicate the findings by Zanto et al. (2016). We discuss the implications of this and all previous experiments below.

## General discussion

Based on theoretical assumptions about the consequences of maintenance of information in WM on delayed memory performance, previous experimental research has investigated the differential effects of encoding memoranda within the context of a simple, slow, and complex span task on immediate and delayed memory. Whereas in the original work on this approach, information from tasks including distraction from the to-be-remembered information seemed to receive a boost for delayed memory performance (McCabe, 2008), others have found alternative explanations, or where unable to replicate the effect. The goal of the present study was to investigate the potential boundary conditions of this so-called McCabe effect. To resolve the ambiguity from previous research on the effect, we asked whether differences in experimental parameters of previous studies (Zanto et al., 2016 vs. Loaiza & Souza, 2021; Souza & Oberauer, 2017) lead to the diverging findings on the McCabe effect. These parameters included (1) Type of Stimuli (doors vs. faces), (2) Type of distractor (pictures vs. math equations), (3) Expectation about task difficulty (mixed vs. blocked lists), (4) Set Size (small vs. large), and (5) Expectation about the LTM test (intentional vs. incidental encoding).

### LTM for complex visual stimuli does not benefit from distraction during encoding

Across four Experiments we were unable to replicate the findings by Zanto et al. (2016) – who showed a benefit from distraction during encoding on delayed memory performance for complex visual stimuli – although most aspects of the study were implemented in the same way. Specifically, across various experimental set-ups, we either found no difference in delayed memory across simple span and distraction conditions (Exps. 2 and 3), or the difference from the WM task transferred to the delayed task (simple > slow > distraction; Exp. 4). Only in Experiment 1, there was evidence for better delayed memory performance in the picture-distraction, compared to simple span task – in case the stimuli were pictures of doors but not faces. However, in this experiment, this benefit was fully explained by the extended amount of time at encoding due to equivalent benefits of the time-matched picture-distraction and slow span condition. Taken together the findings of all four experiments, we provide strong evidence that long-term memory for visual stimuli does not benefit from distraction during encoding. This calls into question the assumptions of the covert retrieval model: Either participants do not covertly retrieve the memoranda from LTM in the presence of a distractor task, or they do so equally for simple, slow span, and distraction conditions. Instead, our results indicate that (at least for complex visual stimuli) a fixed (low) proportion of information is encoded to LTM, which is supported by our findings that in three out of four experiments neither the slow span condition, nor any of the other manipulated variables had a measurable effect on delayed memory performance. Instead the proportion of remembered information in the delayed memory test was equal across all manipulations (see also Bartsch et al., 2019).

This does not mean that people cannot lay down more of those permanent traces into LTM: Previous research has shown that the engagement in elaborative strategies on the content of WM increases later recall (Bartsch et al., 2018, 2022; Loaiza & Lavilla, 2021). Still, the engagement in elaboration would go beyond what McCabe (2008) understood as the covert retrieval of information.

### Time is only beneficial to verbal, not visual WM and LTM

In contrast to Souza and Oberauer (2017), who showed that increased maintenance duration of words improved both immediate as well as delayed memory performance, here we found that the immediate memory of visual material, namely faces and doors, was negatively affected by time: participants recognized more stimuli correctly in the simple compared to the slow span condition in the immediate task. There are two important differences across both studies that could have led to this result: (1) verbal and visual stimuli could differ in their susceptibility to decay and/or (2) the increase in maintenance duration was realized once in-between multiple to be-remembered items (Souza & Oberauer, 2017) but meant a prolonged retention interval following the encoding of a single item in the other case (Zanto et al., 2016).

For the verbal domain evidence generally suggests, that extending an unfilled retention interval after study has no effect on performance (e.g., Oberauer & Lewandowsky, 2016; Ricker & Cowan, 2010; Vallar & Baddeley, 1982), whereas extending the amount of free time *interleaving* items benefits performance (Oberauer, 2022). Within the visual domain evidence is more ambiguous: Some studies show that extending an unfilled retention interval leads to a decline in performance (e.g., Mercer & Duffy, 2015; Ricker et al., 2014; Ricker & Cowan, 2010; Sakai & Inui, 2002), while others have not observed such a detrimental effect (Burke et al., 2015; Gorgoraptis et al., 2011; Kahana & Sekuler, 2002). Our study adds to the literature of the former, showing that immediate memory performance was worse with longer retention intervals.

That time is beneficial to LTM of words both in the scope of simple and complex span tasks has been supported by a recent meta-analysis, which found a small positive effect (Cohen's d ~ 0.2) of maintenance duration on long-term recognition or recall (Hartshorne & Makovski, 2019). Yet, our findings here suggest, that the effect is mostly limited to verbal material, with delayed memory performance for faces being equal across tasks with more or less time (simple vs. slow). Only the stimulus material of doors benefited from longer encoding times in the delayed memory test.

### WM load at encoding does not affect delayed memory

Increasing the set size of to-be-remembered faces had a detrimental effect on the immediate recognition performance in Experiment 3 – yet it did not affect delayed memory performance. This finding is in so far relevant as WM has been theorized to act as gateway into LTM, such that only information successfully stored in WM can be transferred into LTM (Atkinson & Shiffrin, 1968). Thereby, the capacity limit of WM should constrain the acquisition of LTM. Evidence for that prediction so far is mixed: some studies showed that increasing WM load translated into weaker LTM for the same material (Forsberg et al., 2020; Fukuda & Vogel, 2019). Others found no effect of increasing WM load on LTM performance (Bartsch et al., 2019; Krasnoff & Souza, 2021). With Experiment 3 we add to the latter part of the literature – here WM load at encoding did not affect delayed memory.

There are potentially important – yet not systematic – differences between these studies, including the mode of presentation (simultaneous vs. sequential), the domain of memory material (visual vs. verbal); and the memory demand of the test: Bartsch et al. (2019) tested memory for relations between words; Krasnoff and Souza (2021) tested continuous reproduction of color-object conjunctions, and both studies tested all word pairs in the WM test. Fukuda and Vogel (2019) as well as Forsberg et al. (2020) tested item recognition, and only tested a single item in each WM trial. Although the latter might seem as the strongest contender for having caused the differences in previous studies, the present study implemented the exact same parameters in this regard (item recognition of single item, delayed memory test on non-tested items only) and did not find evidence in favor of the gateway hypotheses. The question why these contradicting results occur thereby still remains an open one.

### The effect of intent on delayed memory performance

Popov and Dames (2022) recently provided evidence that the intention to remember at encoding has a strong beneficial effect on long-term memory. Contrary to previous work, they used item-wise instead of list-wise remember instructions, in which only list-items presented in a specific color had to be remembered. Here, we adopted this method to investigate the influence of the intent to remember on the McCabe effect. Our results not only showed that the intent to remember did not influence the occurrence of the McCabe effect, but also showed only weak evidence for an effect of intent in general: Only in the simple span condition, delayed memory performance was improved for items encoded under the remember instruction compared to items encoded under the forget instruction. The reason for this differential effect of the intention to remember between the different task conditions is unclear and we can only speculate about the reasons at this point. One notable difference of our study to that of Popov and Dames (2022) is again that all of their experiments used verbal stimuli, whereas our study used complex visual stimuli. Given that also the amount of time for which information is processed seems to have differential effects for verbal and visual materials, there are reasons to suspect that similar mechanisms might underly the differential effects of intention. However, future research will need to look into these differences more systematically.

## Conclusion

Across four experiments we have shown that delayed memory for faces and other complex visual stimuli does not benefit from covert retrieval during encoding – as suggested to take place during distraction in complex span and Brown Peterson tasks and commonly referred to as the McCabe effect. The transfer of information from WM to LTM does not seem to be influenced by covert retrieval processes. Our data suggests that instead, a fixed proportion of information encoded into LTM is laid down as a more permanent trace.

## Footnotes

1 Imagine a complex span trial of: *king* – distractor – *rose* – distractor – *fish* – distractor – *pony* – distractor – *tooth* – distractor. Following the distractor following the fourth item of the list (“pony”), the participant would need to covertly retrieve the preceding four items back into WM (king, rose, fish, pony).
